# Retrospective identification of pathogenic mycobacterial species in fish: *Mycobacterium pseudoshottsii* YM-3, isolated from a yellowtail fish in 1986 in Kochi, Japan

**DOI:** 10.1128/MRA.00498-23

**Published:** 2023-09-15

**Authors:** Masayuki Imajoh, Shiomi Yoshida, Lisa Nonaka, Yukari Fukushima, Chie Nakajima, Yasuhiko Suzuki, Takayuki Wada

**Affiliations:** 1 Department of Marine Resource Science, Laboratory of Fish Disease, Aquaculture Course, Faculty of Agriculture and Marine Science, Kochi University, Nankoku, Kochi, Japan; 2 Clinical Research Center, National Hospital Organization Kinki-chuo Chest Medical Center, Sakai, Osaka, Japan; 3 Faculty of Human Life Sciences, Shokei University, Kumamoto, Kumamoto, Japan; 4 Division of Bioresources, International Institute for Zoonosis Control, Hokkaido University, Sapporo, Hokkaido, Japan; 5 International Collaboration Unit, Hokkaido University International Institute for Zoonosis Control, Sapporo, Japan; 6 Division of Research Support, Institute for Vaccine Research and Development, Hokkaido University, Sapporo, Hokkaido, Japan; 7 Department of Microbiology, Graduate School of Human Life and Ecology, Osaka Metropolitan University, Osaka, Japan; 8 Osaka International Research Center for Infectious Diseases, Osaka Metropolitan University, Osaka, Japan; University of Southern California, Los Angeles, California, USA

**Keywords:** fish mycobacteriosis, *Mycobacterium pseudoshottsii*, public health

## Abstract

The complete genome sequence of mycobacterial strain YM-3, isolated from cultured yellowtail in 1986, was determined. The strain was *Mycobacterium pseudoshottsii*, a closely related subspecies of *Mycobacterium marinum*, so the strain was isolated earlier than the first report of the subspecies in 2005.

## ANNOUNCEMENT

*Mycobacterium pseudoshottsii*, a subspecies of *Mycobacterium marinum*, can cause mycobacteriosis in both farmed and wild fish (1–5). Genetically, *M. pseudoshottsii* is highly conserved with *M. marinum* and related species, including *Mycobacterium ulcerans*, which is a causative agent of Buruli ulcer (6–9).

In an earlier study of *M. pseudoshottsii* isolates in 2005 (5), Imajoh et al. (10) reported their genetic analysis of bacterial strains isolated from the mycobacteriosis outbreak of cultured yellowtail and striped jack from 1985 to 2004 in Japan, which showed characteristics similar to those of *M. pseudoshottsii*.

**Fig 1:**
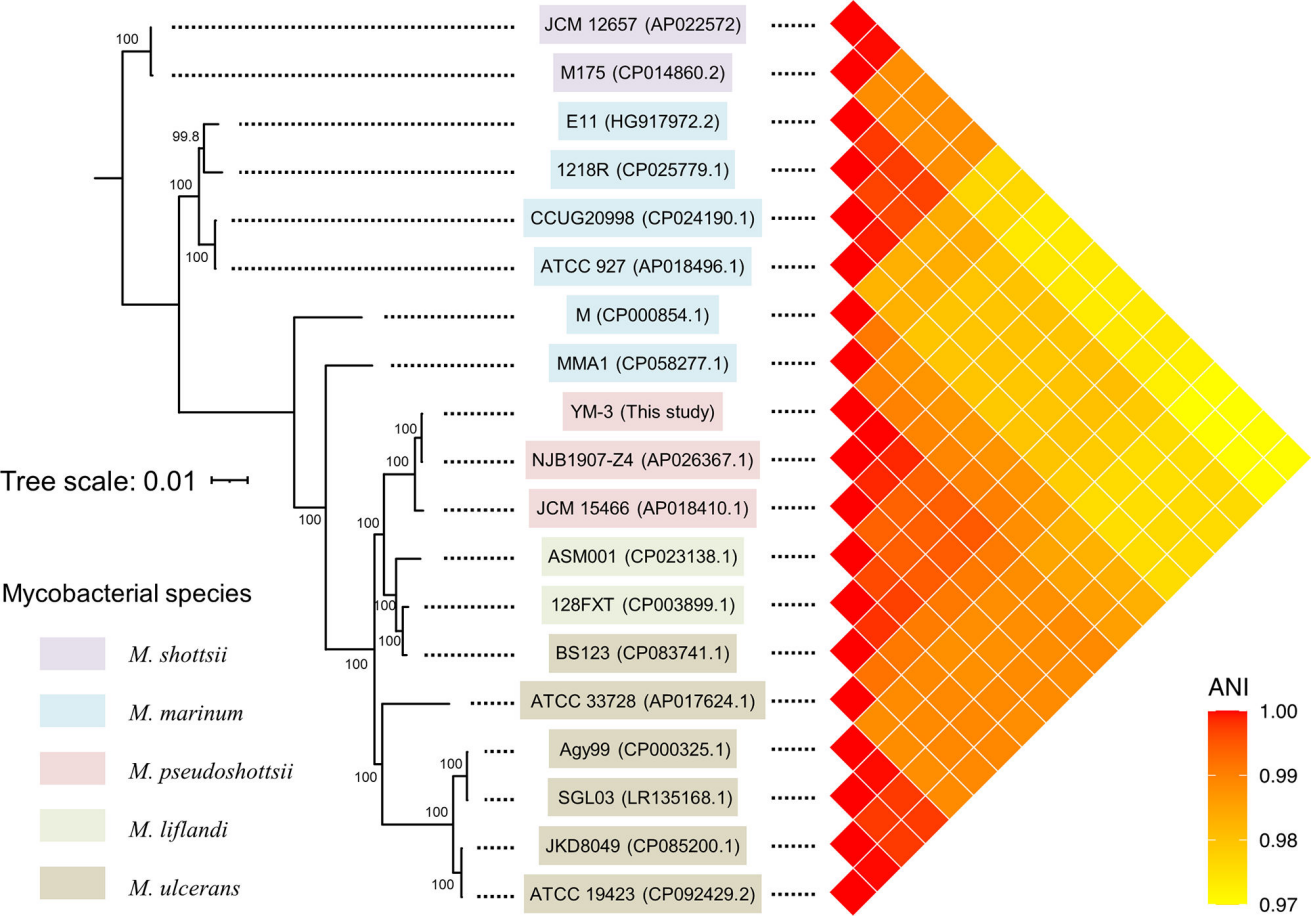
The phylogenetic position of YM-3 was analyzed using 18 complete genome sequences of *M. marinum* complex available in the National Center for Biotechnology Information (NCBI) database. The molecular phylogenetic tree shown at the left was constructed using SNVs retrieved from a rapid core genome multi-alignment tool ParSNP (version 1.2) (15), with the option -x to avoid miscalculation by recombination. The SNV sequences were analyzed using IQ-Tree (version 1.6.12) (16) for construction of the maximum likelihood tree with 1,000 times iteration for bootstrapping. The model was selected by BIC using the option -m MFP + AFC to find the best model (TMV + F + ASC). Two strains of *Mycobacterium shottsii* (JCM 12657 and M175) were used as the outgroup. The bootstraps were shown at each branch. The respective species designations were based on the information registered with NCBI. The pairwise gANI scores between two sequences are represented by the color at the right.

In this study, the complete genome sequence of the YM-3 strain, one of the oldest strains reported by Imajoh et al. (10), was sequenced using high-throughput sequencers. Genomic DNA was purified from a colony of frozen stock after it was cultured on Ogawa solid media (Kyokuto Pharmaceutical Industrial, Japan) at 25°C for 2 wk using a method described in a previous study (11). Using PacBio RSII (Pacific Biosciences, USA), 116,422,661 bp consisting of 15,852 highly accurate long reads (7,338 bp of average length, N_50_ of 10,111 bp) were obtained, and libraries were constructed using SMRTbell Template Prep Kit v1.0 after size selection between 4,000 and 50,000 bp by BluePippin (Sage Science, USA). We also obtained 714,998,085 bp consisting of 1,214,751 paired-end reads using a TruSeq DNA PCR-Free Kit and MiSeq Sequencing Kit v3 (600 cycles) (Illumina, USA) and assembled them using Unicycler (version 0.4.3) (12) after validating the sequence quality with the “stats” command of Seqkit v. 2.0.0 (13). Consequently, one circularized sequence (6,050,184 bp) was obtained as a complete genome, with read data coverage of 113x. To compare their genome sequences, a phylogenetic tree of the core genome was constructed, and genomic average nucleotide identity (gANI) was measured pairwise using FastANI (14) (Fig. 1). Results showed that YM-3 and two *M. pseudoshottsii* strains were almost identical, with gANI exceeding 0.997, differentiating them as the same subspecies. Default parameters were used for all software, unless otherwise specified in the analyses.

Auto-annotation with DFAST v. 1.2.0 (17) resulted in the identification of 5,531 protein-coding sequences (CDSs). For the open reading frame structural annotation of CDS, the “Prodigal (18)” option was selected. The complete genome was confirmed to be rotated and flipped, with *dnaA* located at the 5′ end of the sequence using a tool function. IS2404 had a confirmed insertion of 176 copies in the genome (Table 1), whereas no IS2606 was found, as were the two strains of *M. pseudoshottsii* (JCM 15466 and NJB1907-Z4).

**TABLE 1:** Features of the complete genome sequences of *M. pseudoshottsii* strains

Strain	YM-3	NJB1907-Z4	JCM 15466
Length (bp)	6,050,184	6,051,062	6,061,597
GC%	65.6	65.6	65.6
No. of contig	1	1	1
No. of CDS*^a^*	5,531	5,535	5,555
No. of IS2404	176	176	180
No. of IS2606	0	0	0
Genbank accession no.	AP028092.1	AP026367.1	AP018410.1
Reference	This study	Komine et al. (19)	Yoshida et al. (20)

^a^ All three genome sequences were annotated using DFAST (ver. 1.2.0) to harmonize the comparison.

The putative plasmid of YM-3 was not assembled, although genes involved in mycolactone production were previously detected through PCR (10). Similarly, a genome sequence without a plasmid sequence was reported for the type strain (JCM 14566) of this organism (20). The genome sequence of NJB1907-Z4 contained a partial plasmid sequence in which the mycolactone polyketide synthase gene was not found (19). This plasmid may be unstable under artificial culture conditions; even if it were retained, it would be difficult to decipher the sequence (21).

## Data Availability

The genome sequence of *M. pseudoshottsii* YM-3 was deposited in INSDC via DDBJ under accession number AP028092. Raw sequence data are available in the DDBJ Sequence Read Archive (DRA) under DRA accession numbers DRR488391 (MiSeq) and DRR488392 (PacBio).

## References

[1] Komine T, Ihara H, Ono K, Yoshida M, Sugimoto Y, Inohana M, Fukano H, Kurata O, Wada S. 2022. A case of mycobacteriosis associated with Mycobacterium pseudoshottsii in aquarium-reared fish in Japan. J Vet Med Sci 84:1617–1620. doi:10.1292/jvms.22-0318 36273872 PMC9791231

[2] Mugetti D, Varello K, Gustinelli A, Pastorino P, Menconi V, Florio D, Fioravanti ML, Bozzetta E, Zoppi S, Dondo A, Prearo M. 2020. Mycobacterium pseudoshottsii in mediterranean fish farms: new trouble for European aquaculture? Pathogens 9:610. doi:10.3390/pathogens9080610 32726963 PMC7459456

[3] Nakanaga K, Hoshino Y, Hattori Y, Yamamoto A, Wada S, Hatai K, Makino M, Ishii N. 2012. Mycobacterium pseudoshottsii isolated from 24 farmed fishes in Western Japan. J Vet Med Sci 74:275–278. doi:10.1292/jvms.11-0226 21986278

[4] Stine CB, Jacobs JM, Rhodes MR, Overton A, Fast M, Baya AM. 2009. Expanded range and new host species of Mycobacterium shottsii and M. pseudoshottsii. J Aquat Anim Health 21:179–183. doi:10.1577/H09-005.1 20043404

[5] Rhodes MW, Kator H, McNabb A, Deshayes C, Reyrat JM, Brown-Elliott BA, Wallace R, Trott KA, Parker JM, Lifland B, Osterhout G, Kaattari I, Reece K, Vogelbein W, Ottinger CA. 2005. Mycobacterium pseudoshottsii sp. nov., a slowly growing chromogenic species isolated from chesapeake bay striped bass (Morone saxatilis). Int J Syst Evol Microbiol 55:1139–1147. doi:10.1099/ijs.0.63343-0 15879246

[6] Hikima J-I, Sakai M, Aoki T, Takeyama H, Hawke J, Mori K, Tashiro K, Kuhara S. 2016. Draft genome sequence of the fish pathogen Mycobacterium pseudoshottsii strain JCM15466, a species closely related to M. marinum. Genome Announc 4:e01630-15. doi:10.1128/genomeA.01630-15 26868383 PMC4751307

[7] Tobias NJ, Doig KD, Medema MH, Chen H, Haring V, Moore R, Seemann T, Stinear TP. 2013. Complete genome sequence of the frog pathogen Mycobacterium ulcerans ecovar Liflandii. J Bacteriol 195:556–564. doi:10.1128/JB.02132-12 23204453 PMC3554023

[8] Stinear TP, Seemann T, Pidot S, Frigui W, Reysset G, Garnier T, Meurice G, Simon D, Bouchier C, Ma L, Tichit M, Porter JL, Ryan J, Johnson PDR, Davies JK, Jenkin GA, Small PLC, Jones LM, Tekaia F, Laval F, Daffé M, Parkhill J, Cole ST. 2007. Reductive evolution and niche adaptation inferred from the genome of Mycobacterium ulcerans, the causative agent of Buruli ulcer. Genome Res 17:192–200. doi:10.1101/gr.5942807 17210928 PMC1781351

[9] Pidot SJ, Asiedu K, Käser M, Fyfe JAM, Stinear TP. 2010. Mycobacterium ulcerans and other mycolactone-producing mycobacteria should be considered a single species. PLoS Negl Trop Dis 4:e663. doi:10.1371/journal.pntd.0000663 20668542 PMC2910673

[10] Imajoh M, Sugiura H, Hashida Y, Hatai K, Oshima S, Daibata M, Kawai K. 2013. Genotypic characteristics of a Mycobacterium sp. isolated from yellowtail Seriola quinqueradiata and striped Jack Pseudocaranx dentex in Japan. Microbiol Immunol 57:13–20. doi:10.1111/j.1348-0421.2012.00514.x 23043488

[11] Belisle JT, Sonnenberg MG. 1998. Isolation of genomic DNA from mycobacteria. Methods Mol Biol 101:31–44. doi:10.1385/0-89603-471-2:31 9921467

[12] Wick RR, Judd LM, Gorrie CL, Holt KE. 2017. Unicycler: resolving bacterial genome assemblies from short and long sequencing reads. PLoS Comput Biol 13:e1005595. doi:10.1371/journal.pcbi.1005595 28594827 PMC5481147

[13] Shen W, Le S, Li Y, Hu F. 2016. SeqKit: a cross-platform and ultrafast toolkit for FASTA/Q file manipulation. PLoS One 11:e0163962. doi:10.1371/journal.pone.0163962 27706213 PMC5051824

[14] Jain C, Rodriguez-R LM, Phillippy AM, Konstantinidis KT, Aluru S. 2018. High throughput ANI analysis of 90K prokaryotic genomes reveals clear species boundaries. Nat Commun 9:5114. doi:10.1038/s41467-018-07641-9 30504855 PMC6269478

[15] Treangen TJ, Ondov BD, Koren S, Phillippy AM. 2014. The harvest suite for rapid core-genome alignment and visualization of thousands of Intraspecific microbial genomes. Genome Biol 15:524. doi:10.1186/s13059-014-0524-x 25410596 PMC4262987

[16] Nguyen L-T, Schmidt HA, von Haeseler A, Minh BQ. 2015. IQ-TREE: a fast and effective stochastic algorithm for estimating maximum-likelihood phylogenies. Mol Biol Evol 32:268–274. doi:10.1093/molbev/msu300 25371430 PMC4271533

[17] Tanizawa Y, Fujisawa T, Nakamura Y. 2018. DFAST: a flexible prokaryotic genome annotation pipeline for faster genome publication. Bioinformatics 34:1037–1039. doi:10.1093/bioinformatics/btx713 29106469 PMC5860143

[18] Hyatt D, Chen GL, Locascio PF, Land ML, Larimer FW, Hauser LJ. 2010. Prodigal: prokaryotic gene recognition and translation initiation site identification. BMC Bioinformatics 11:119. doi:10.1186/1471-2105-11-119 20211023 PMC2848648

[19] Komine T, Fukano H, Yoshida M, Inohana M, Hoshino Y, Kurata O, Wada S. 2022. Complete genome and partial megaplasmid sequences of Mycobacterium pseudoshottsii strain NJB1907-Z4,isolated from an aquarium-reared Japanese sardine (Sardinops melanostictus) in Japan. Microbiol Resour Announc 11:e0078522. doi:10.1128/mra.00785-22 36350130 PMC9753711

[20] Yoshida M, Miyamoto Y, Ogura Y, Hayashi T, Hoshino Y. 2017. Complete chromosome sequence of a mycolactone-producing mycobacterium, Mycobacterium pseudoshottsii. Genome Announc 5:e01363-17. doi:10.1128/genomeA.01363-17 29192083 PMC5722069

[21] Pidot SJ, Tobias NJ, Stinear T. 2009. The pMUM megaplasmid of *Mycobacterium ulcerans* and closely related mycobacteria: a blueprint for the synthesis of mycolactones, p 283–296. In Schwartz E (ed), Microbial megaplasmids. Springer: Berlin, Heidelberg. doi:10.1007/978-3-540-85467-8

